# Good Laboratory Practices: Myers et al. Respond

**DOI:** 10.1289/ehp.0900884R

**Published:** 2009-11

**Authors:** John Peterson Myers, Frederick S. vom Saal, Julia A. Taylor, Benson T. Akingbemi, Koji Arizono, Scott Belcher, Theo Colborn, Ibrahim Chahoud

**Affiliations:** Environmental Health Sciences, Charlottesville, Virginia, E-mail: jpmyers@ehsic.org; Division of Biological Science, University of Missouri, Columbia, Missouri; Department of Anatomy, Physiology & Pharmacology, College of Veterinary Medicine, Auburn University, Auburn, Alabama; Faculty of Environmental and Symbiotic Science, Prefectural University of Kumamoto, Tsukide, Kumamoto, Japan; Department of Pharmacology & Cell Biophysics, Center for Environmental Genetics, University of Cincinnati, Cincinnati, Ohio; The Endocrine Disruption Exchange, Paonia, Colorado; Institut für Klinische Pharmakologie und Toxikologie Charité–Universitätsmedizin Berlin, Campus Benjamin Franklin, Berlin, Germany

We are in complete agreement with the statement by Becker et al. that “having confidence in scientific procedures and data is the *sine qua non* for determining the safety of chemicals and chemical products.” Our aim in writing the commentary (Myers et al. 2009) was not to challenge the original intent of Good Laboratory Practices (GLP) requirements, which was to establish standards of record keeping in contract laboratory research so as to reduce the likelihood of fraud. Our goal instead was to show—through an analysis of the application of GLP data on bisphenol A (BPA) in regulatory proceedings—that GLP by itself is insufficient to guarantee valid and reliable science. Becker et al. appear to have missed the point of our commentary entirely.

In the case of BPA, three GLP studies have been offered by industry-sponsored laboratories as proof of the chemical’s safety (Cagen et al. 1999; Tyl et al. 2002, 2008). Each has errors in study design and/or data interpretation that are sufficiently serious as to invalidate the conclusions of these studies (Myers et al. 2009). Nevertheless, because the studies were conducted using GLP guidelines, they were judged by regulators as being more reliable than the many National Institutes of Health (NIH)-funded and peer-reviewed studies that have reported adverse effects (Richter et al. 2007;vom Saal et al. 2007).

As our commentary (Myers et al. 2009) clearly establishes, GLP did not guarantee the scientific validity of these three studies. Because previous analyses had identified serious flaws in the first two of those GLP studies, we focused critical attention on the most recent (Tyl et al. 2008), which both the European Food Safety Authority (EFSA 2006) and the U.S. Food and Drug Administration (FDA) had identified as key in their BPA risk assessments (FDA 2008). We found three main flaws: *a*) the animals were inexplicably insensitive to estrogen; *b*) the assays were outdated and insensitive compared with methods used in NIH-funded research showing adverse effects; and *c* ) validity of the findings was challenged. For example, the prostate weights of control animals reported by Tyl et al. (2008) were > 70% larger (mean, > 72 mg) than those reported by numerous laboratories, including a previously published study using CD-1 mice [conducted at RTI, where the study by Tyl et al. (2008) was conducted] that reported mean prostate weights of 46 mg in CD-1 males that were examined at a similar age (Heindel et al. 1995).

Since we published our commentary (Myers et al. 2009), a possible contributor to both the estrogen insensitivity and the enlarged control prostates has been suggested: Approximately 3 years before the experiments that formed the basis of the study by Tyl et al. (2008), there was a polycarbonate fire that released BPA into the RTI laboratory where the research was conducted (Kissinger and Rust 2009). An investigation revealed that animals in the laboratory were exposed to low doses of BPA that government-funded science (Richter et al. 2007) indicates could affect research animals.

Additional uncertainties about Tyl et al.’s study (Tyl et al. 2008) have now been identified by the lead author. Whereas the published paper reports that the animals were examined at approximately 14 weeks of age, Tyl testified at an FDA hearing in September 2008 that they were 6 months of age, and then at a German Environmental Protection Agency hearing in March 2009 that they were 5 months of age (Kissinger and Rust 2009). There she confirmed that the information in the original article was inaccurate. Because an animal’s physiology changes as it ages, these contradictory statements are problematic for all reported outcomes; even at 5–6 months of age, normal, healthy CD-1 male mice would not have the grossly enlarged prostates reported by Tyl et al. (2008).

The use of flawed science, however, is not the only concern. The type of multigeneration testing approach used in these studies is, quite simply, insufficient for the testing of endocrine-disrupting chemicals. This is not a new concept. The need for more specific tests for endocrine-active compounds led in 1998 to the establishment at the U.S. Environmental Protection Agency (U.S. EPA) of the Endocrine Disruptor Screening Program, mandated by Congress (U.S. EPA 1998). After virtually no progress for over a decade, in 2009 the U.S. EPA finally announced a set of testing procedures that will be examined. The proposed “new” methodology, heavily dependent upon traditional toxicologic methods used in multigenerational GLP studies, is still woefully inadequate (Colborn 2009).

The letter by Becker et al. provides a striking example of the reluctance of industry lobbyists to hear this message. In the eyes of the 36 scientific colleagues who coauthored our commentary (Myers et al. 2009), the BPA studies that Becker et al. attempt to defend are so seriously flawed as to be indefensible. Rather than continue to defend a dead issue, we encourage industry representatives to come into the 21st century and help us devise new paradigms for testing endocrine-disrupting chemicals that will safeguard human health.
